# Supplementary material for: Phosphatidylethanolamines Are Associated with Nonalcoholic Fatty Liver Disease (NAFLD) in Obese Adults and Induce Liver Cell Metabolic Perturbations and Hepatic Stellate Cell Activation
Source: Int J Mol Sci. 2023 Jan 5;24(2):1034. doi: 10.3390/ijms24021034 (PMC9861886; doi:10.3390/ijms24021034)
Supplement: Supplementary file 1 [file ijms-24-01034-s001.zip › ijms-2114318-supplementary.pdf]

Phosphatidylethanolamines Are Associated with Nonalcoholic Fatty Liver Disease (NAFLD) in Obese Adults and Induce Liver Cell Metabolic Perturbations and Hepatic Stellate Cell Activation

Samaa Shama [1,2], Hyejeong Jang [3,4], Xiaokun Wang [1], Yang Zhang [1], Nancy Nabil Shahin [5], Tarek Kamal Motawi [5,*], Seongho Kim [3,4], Samer Gawrieh [6] and Wanqing Liu [1,4,7,*]

[1] Department of Pharmaceutical Sciences, Eugene Applebaum College of Pharmacy and Health Sciences, Wayne State University, Detroit, MI 48201, USA
[2] Cell-Based Analysis Unit, Reference Laboratory, Egyptian Drug Authority, Cairo 12618, Egypt
[3] Biostatistics and Bioinformatics Core, Department of Oncology, Karmanos Cancer Institute,
Wayne State University, Detroit, MI 48201, USA
[4] Department of Oncology, Wayne State University School of Medicine, Detroit, MI 48201, USA
[5] Department of Biochemistry, Faculty of Pharmacy, Cairo University, Cairo 11562, Egypt
[6] Division of Gastroenterology and Hepatology, Department of Medicine, Indiana University School of
Medicine, Indianapolis, IN 46202, USA
[7] Department of Pharmacology, Wayne State University School of Medicine, Detroit, MI 48201, USA
***** Correspondence: tarek.motawi@pharma.cu.edu.eg (T.K.M.); wliu@wayne.edu (W.L.);
Tel.: +20-122-313-8667 (T.K.M.); +1-313-577-3375 (W.L.)

Table S1. List of gene-specific primer pairs.

Gene	Primer sequence
GAPDH	*Forward: 5’-GTCTCCTCTGACTTCAACAGCG- 3’*
	Reverse: 5’-ACCACCCTGTTGCTGTAGCCAA- 3’
CPT	*Forward: 5’-TCCAGAGTCCGATTGATTTTTGC- 3’*
	Reverse: 5’-TCCAGTTGGCTTATCGTGGTG- 3’
PPARα	*Forward: 5’-TCGGCGAGGATAGTTCTGGAAG-3’*
	Reverse: 5’-GACCACAGGATAAGTCACCGAG- 3’
DGAT1	*Forward: 5’- CAATCTGACCTACCGCGATCT - 3’*
	Reverse: 5’- TCGATGATGCGTGAGTAGTCC - 3’
FASN	*Forward: 5’-CCGAGACACTCGTGGGCTA- 3’*
	Reverse: 5’-CTTCAGCAGGACATTGATGC- 3’
SREBP	*Forward: 5’-ACTTCTGGAGGCATCGCAAGCA- 3’*
	Reverse: 5’-AGGTTCCAGAGGAGGCTACAAG- 3’
FXR	*Forward: 5’-GGTCTCGTAGACGAAGGACTGA- 3’*
	Reverse: 5’-TGTCTGCTCTGAGACTCAGCTC- 3’
LC3	*Forward: 5’- GCAGCCTTTGTTCCAGAGAC- 3’*
	Reverse: 5’- CTGGAAAAGTGGAGGCTGAG- 3’
mTOR	*Forward: 5’-GCAGATTTGCCAACTATCTTCGG- 3’*
	Reverse: 5’-CAGCGGTAAAAGTGTCCCCTG- 3’
BAX	*Forward: 5’-CTGCAGAGGATGATTGCCG-3’*
	Reverse: 5’-TGCCACTCGAAAAAGACCT-3’
PAK2	*Forward: 5’-CACCCGCAGTAGTGACAGAG- 3’*
	Reverse: 5’-GGGTCAATTACAGACCGTGTG- 3’
CYCs	*Forward: 5’-GAGCGGGAGTGTTCGTTGT-3’*
	Reverse: 5’- GTCTGCCCTTTCTTCCTTCT-3’
BCL2	*Forward: 5’- TCCCTCGCTGCACAAATA CTC-3’*
	Reverse: 5’- ACGACCCGATGGCCATAGA-3’
COL1A1	*Forward: 5’- GATTCCCTGGACCTAAAGGTGC- 3’*
	Reverse: 5’- AGCCTCTCCATCTTTGCCAGCA- 3’

COL3A1	*Forward: 5'- TGGTCTGCAAGGAATGCCTGGA- 3'*
	Reverse: 5'- TCTTTCCCTGGGACACCATCAG- 3'
TIMP1	*Forward: 5'- GGAGAGTGTCTGCGGATACTTC- 3'*
	Reverse: 5'- GCAGGTAGTGATGTGCAAGAGTC- 3'
TIMP3	*Forward: 5'- TACCGAGGCTTCACCAAGATGC- 3'*
	Reverse: 5'- CATCTTGCCATCATAGACGCGAC- 3'
TGFβ	*Forward: 5'- TACCTGAACCCGTGTTGCTCTC- 3'*
	Reverse: 5'- GTTGCTGAGGTATCGCCAGGAA- 3'
TNFα	*Forward: 5'- CCCGAGTGACAAGCCTGTAG- 3'*
	Reverse: 5'- GATGGCAGAGAGGAGGTTGAC- 3'
IL6	*Forward: 5'- ACTCACCTCTTCAGAACGAATTG- 3'*
	Reverse: 5'- CCATCTTTGGAAGGTTCAGGTTG- 3'
